# Induction of Trained Innate Immunity in Human Monocytes by Bovine Milk and Milk-Derived Immunoglobulin G

**DOI:** 10.3390/nu10101378

**Published:** 2018-09-27

**Authors:** Marloes van Splunter, Thijs L. J. van Osch, Sylvia Brugman, Huub F. J. Savelkoul, Leo A. B. Joosten, Mihai G. Netea, R. J. Joost van Neerven

**Affiliations:** 1 Cell Biology and Immunology, Wageningen University, Wageningen, P.O. Box 338, 6708 WD Wageningen, The Netherlands; marloes.vansplunter@gmail.com (M.v.S.); thijsvanosch@live.nl (T.L.J.v.O.); sylvia.brugman@wur.nl (S.B.); huub.savelkoul@wur.nl (H.F.J.S.); 2 Department of Internal Medicine and Radboud Center for Infectious Diseases (RCI), Radboud University Medical Center, 6525 GA Nijmegen, The Netherlands; Leo.Joosten@radboudumc.nl (L.A.B.J.); Mihai.Netea@radboudumc.nl (M.G.N.); 3 Department for Genomics & Immunoregulation, Life and Medical Sciences Institute (LIMES), University of Bonn, 53115 Bonn, Germany; 4 FrieslandCampina, 3818 LE Amersfoort, The Netherlands

**Keywords:** innate immune memory, trained immunity, raw bovine milk, bovine IgG, bovine lactoferrin, dietary compounds, monocytes

## Abstract

Innate immune memory, also termed “trained immunity” in vertebrates, has been recently described in a large variety of plants and animals. In most cases, trained innate immunity is induced by pathogens or pathogen-associated molecular patterns (PAMPs), and is associated with long-term epigenetic, metabolic, and functional reprogramming. Interestingly, recent findings indicate that food components can mimic PAMPs effects and induce trained immunity. The aim of this study was to investigate whether bovine milk or its components can induce trained immunity in human monocytes. To this aim, monocytes were exposed for 24 h to β-glucan, Toll-like receptor (TLR)-ligands, bovine milk, milk fractions, bovine lactoferrin (bLF), and bovine Immunoglobulin G (bIgG). After washing away the stimulus and a resting period of five days, the cells were re-stimulated with TLR ligands and Tumor necrosis factor (TNF-) and interleukin (IL)-6 production was measured. Training with β-glucan resulted in higher cytokine production after TLR1/2, TLR4, and TLR7/8 stimulation. When monocytes trained with raw milk were re-stimulated with TLR1/2 ligand Pam3CSK4, trained cells produced more IL-6 compared to non-trained cells. Training with bIgG resulted in higher cytokine production after TLR4 and TLR7/8 stimulation. These results show that bovine milk and bIgG can induce trained immunity in human monocytes. This confirms the hypothesis that diet components can influence the long-term responsiveness of the innate immune system.

## 1. Introduction

The immune system is divided into two arms, the innate immune system and the adaptive immune system, of which only the latter is known to build long-lasting immune memory in T and B cells. However, recent observations have revealed that the innate immune system can also adapt to previous insults and develop a non-specific memory after infections, a process termed “trained innate immunity” [1,2]. The concept of trained immunity is based on the observation that after a primary infection, an enhanced innate immune response is induced in response to secondary infection or stimulation. In contrast to adaptive immune memory, this enhanced secondary response of trained innate immune cells is not only specific for the antigen that induced the primary response, but is rather a non-specific enhanced response to heterologous stimuli [1]. It is known that invertebrates respond better towards secondary infections, both to the same pathogen as well as towards other unrelated infections. It has been shown that in plants and invertebrates, processes termed “systemic acquired resistance” and “immune priming” occur widely in organisms that possess only an innate immune system [3,4,5,6,7,8,9]. Interesting from an evolutionary perspective, among vertebrates there are indications that trained immunity occurs in teleost fish, which are the first vertebrates having a functioning adaptive and innate immune system, as reviewed by Petit and Wiegertjes [10].

In humans, evidence for the existence of trained immunity first emerged from epidemiological studies on vaccination responses, which have indicated that vaccination induces protection not only against the target disease, but also cross-protection against other pathogens [11]. The best known example of this cross-protection is seen after Bacillus Camette-Guérin (BCG) vaccination against *Mycobacterium tuberculosis*, which was shown to protect against all-cause mortality by reducing neonatal sepsis, respiratory infection, and fever [12]. Furthermore, it was shown that BCG vaccination in humans induced trained immunity in both monocytes and Natural killer (NK) cells three months after vaccination, which was mediated by increased H3K4 trimethylation in monocytes [13,14].

The mechanism of trained immunity was first described by Quintin et al., who showed that mice survived when treated first with a sublethal dose of *Candida albicans* followed by a secondary lethal *C. albicans* infection [15]. This outcome was found both in wild-type mice and in T/B-cell defective (Rag 1-deficient) mice, indicating that the adaptive immune system was not involved in the induction of trained immunity. Subsequently, it was shown that β-glucan derived from *C. albicans* could induce trained immunity in purified human monocytes [15]. Some types of β-glucans are present in the cell wall of *C. albicans*, while other types are also present in food as mushrooms, baker’s and brewer’s yeast, and the cell walls of plants including wheat and oat, as reviewed by Meena et al. [16]. These dietary β-glucans may induce trained immunity as well, although this remains to be demonstrated.

Low-density lipoprotein (oxLDL) particles, induced in blood as a result of Western diets, are known to induce trained immunity in human cells in vitro [17]. A Western diet resulted in transient systemic inflammatory responses in mice, yet at the same time induce long-lived epigenetic and transcriptomic reprogramming of granulocyte–monocyte progenitor cells, leading to trained immunity by monocytes [18]. It can thus be concluded that not only infections by pathogens or vaccination can induce trained immunity, but also dietary components.

Epidemiological studies have revealed that children growing up on a farm and consuming (raw) farm milk have a reduced incidence of asthma, atopy, hay fever, respiratory tract infections (RTI), and otitis media compared to children that consumed heat-treated milk [19,20,21]. The components that may cause the reduction of allergy and infections in children are thus milk processing-sensitive, and are therefore thought to be heat-sensitive milk proteins. These findings indicate that raw milk or its components can modify immune responses in vivo.

More than 400 components have been identified in bovine milk, and these can be subdivided in multiple fractions [22]. Bovine milk is composed of water (87%), lactose (4–5%), protein (whey and casein) (3%), lipids and fat (3–4%), minerals (0.8%), and vitamins (0.1%) [23,24,25,26]. Whey proteins make up 20% of protein concentration of milk, whereas caseins represent 80% of milk proteins [27]. The most abundant whey proteins are β-lactoglobulin, α-lactalbumin, immunoglobulins, such as bovine Immunoglobulin G (bIgG), serum albumin, and lactoferrin (bLF) [27]. Immunologically, the best studied whey proteins are bovine IgG and lactoferrin [28,29,30,31].

The aim of this study was to study whether raw bovine milk, milk fractions, or milk proteins such as lactoferrin and IgG can induce trained immunity in human monocytes. 

## 2. Materials and Methods

### 2.1. Blood Samples and Monocyte Purification

Buffy coats were collected from healthy blood donors at the Sanquin Blood Supply in Nijmegen, the Netherlands. Human peripheral blood mononuclear cells (PBMCs) were isolated from buffy coats using Ficoll plaque plus (17-1440-02, GE Healthcare Life Sciences, Uppsala, Sweden). Monocytes were enriched from freshly isolated PBMCs using negative selection with an Easysep human mono-enrichment kit, according to manufacturer’s protocol (19359, Stemcell Technologies, Köln, Germany). Purity was tested by flow cytometry staining isolated cells with α-CD14 (555397, BD Pharmingen, Franklin Lakes, NJ, USA), α-CD3 (555334, BD Pharmingen, Franklin Lakes, NJ, USA), α-CD19 (562947 BD Horizon, Franklin Lakes, NJ, USA), α-CD56 (555516, BD Pharmingen), and fixable viability dye eFluor 450 (65-0863-14, eBioscience, San Diego, CA, USA). Stained cells were measured on FACS CANTO II. Monocytes isolated from subjects used for analysis all had a purity of >70% CD14^+^ monocytes and <4% CD3^+^ T cells (see Supplementary Figure S1). 

### 2.2. Trained Immunity Model in Human Monocytes

**Figure 1:**
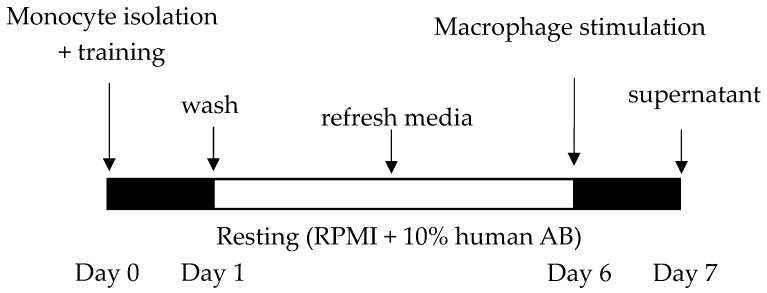
Trained immunity model in human monocytes. At day 0, peripheral blood mononuclear cells (PBMCs) were isolated from buffy coats, and monocytes were isolated by negative selection. Monocytes were stimulated (trained) for 24 h, after which the stimulus was washed away and monocytes differentiated towards macrophages during a five-day resting period. At day six, macrophages were re-stimulated with Toll-like receptor (TLR)-ligands, and after 24 h the supernatant was collected and measured for cytokines.

The trained immunity model in human monocytes was performed as previously described [32,33] with some adjustments, and is depicted in Figure 1. This in-vitro model is widely accepted as reflecting trained immunity in macrophages [1,2,3,4,5,6]. Isolated monocytes were transferred into a 96-well plate (1 × 10^5^ monocytes/well) (Costar3596, Washington, DC, USA), and the training stimulation or culture medium Roswell Park Memorial Institute (RPMI) 1640 Dutch modifications from Sigma-Aldrich (St. Louis, MO, USA), supplemented with 1% gentamicin, 1% l-glutamine, and 1% pyruvate (Life Technologies, Nieuwerkerk, The Netherlands), but without serum, was added in a total volume of 200 μL for 24 h at 37 °C. For the different training stimuli and their concentrations, see Section 2.3. After 24 h, the plates were washed twice with warm Phosphate-buffered saline (PBS), and RPMI medium +10% human pooled serum was added for five days and refreshed after two to three days. In these five days, monocytes differentiated towards macrophages, and at day six macrophages were stimulated in the presence or absence of TLR-ligands Pam3CSK4 10 μg/mL (L2000; EMC microcollections, Tübingen, Germany) Ultra-pure lipopolysaccharide (LPS) 0.1 μg/mL (3pelps, Invivogen, San Diego, CA, USA) and R848 10 μg/mL (TLRL-R848-5, Invivogen). After 24 h of secondary stimulation, the supernatant was collected and stored at −80 °C.

### 2.3. Reagents

*Candida albicans* β-glucan (1 μg/mL) was a kind gift of Prof. David Williams (East Tennessee State University), and was isolated and purified as previously described [34]. Pooled human serum was collected in serum tubes and heat inactivated for 30 min at 56 °C. Afterwards, the serum was aliquoted and stored −80 °C until use. For every refreshment of the medium, freshly pooled serum was thawed. 

Raw bovine milk (1:100), bovine lactoferrin (100 μg/mL), bovine IgG (200 μg/mL), lactose (463 μg/mL), whey protein (liquid SPC 1:100), casein (264 μg/mL), cream serum (3.6 μg/mL), and anhydrous milk fat (458.9 μg/mL) were obtained from FrieslandCampina, Wageningen, The Netherlands. As bLF and bIgG contained some endotoxin, they were treated with Triton-X114 to remove LPS, as described by Teodorowicz et al. [35]. After LPS removal, bIgG and bLF contained less than 10 pg/mL LPS at the dilutions used for all experiments. Endotoxin levels were measured using an Endozyme recombinatant Factor C assay (Hyglos, Bernried, Germany).

### 2.4. Cytokine Measurement

In the supernatant, the production of interleukin (IL)-6 (558276, BD Pharmingen, Franklin Lakes, NJ, USA) and Tumor necrosis factor (TNF)-α (560112, BD Pharmingen) was measured using cytometric bead array on the FACS CANTO II, according to manufacturer’s protocol.

### 2.5. Statistical Analysis

**Figure 2:**
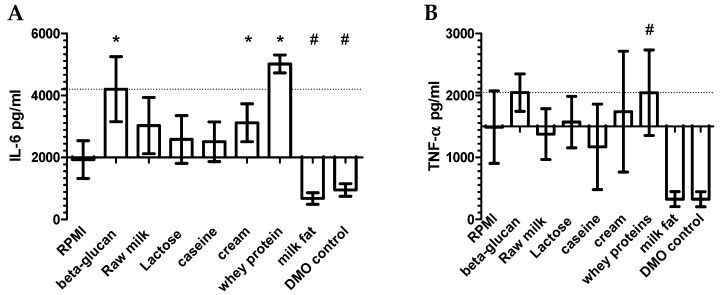
Selection of bovine milk fractions that can induce trained immunity. Monocytes were stimulated 24 h in the presence or absence of β-glucan (1 μg/mL), raw milk (1:100), lactose (463 μg/mL), casein (264 μg/mL), cream (3.6 μg/mL), whey protein (liquid serum protein concentrate (SPC) 1:100), milk fat dissolved in Dimethyl sulfoxide (DMSO) (458.9 μg/mL), and DMSO (1.8 uL/mL, the same concentration as used for milk fat). After five days of rest, the differentiated macrophages were stimulated for 24 h with R848 (10 μg/mL). After stimulation with R848, the produced IL-6 and Tumor necrosis factor (TNF)-α (pg/mL) were measured in supernatant. Data shown as mean ± standard error of mean (SEM), with the IL-6 and TNF-α production of non-trained cells (Roswell Park Memorial Institute (RPMI) medium) as the *x*-axis and the cytokine production by β-glucan trained cells (positive control) as dotted lines. RPMI, Beta-glucan, raw milk, lactose: *n* = 5; all other stimuli: *n* = 3. Statistical analysis was done by performing a paired T-test. * *p* < 0.05, # *p* < 0.10.

IBM SPSS Statistics software, version 23, Armonk, NY, USA, was used to perform statistical analysis. All experiments with β-glucan, raw bovine milk, bovine lactoferrin and bovine IgG were performed at least five times, with monocytes isolated from PBMC, with a minimum of eleven volunteers in total. In order to assess the training effect of a specific ligand upon a secondary stimulation, non-trained cells stimulated with Pam3CSK4, LPS or R848 were compared with trained cells re-stimulated with Pam3CSK4, LPS, or R848. Differences between the groups were analyzed using the Wilcoxon signed-rank test, and were considered statistically significant at a *p* value of <0.05. On the screening experiments of the training stimuli (Figure 2 and Supplementary Figure S2), statistical analysis was performed using a paired T-test, after checking the measurements for being normally distributed using a Shapiro Wilk test.

## 3. Results

### 3.1. Screening of Bovine Milk and Milk Fractions for the Induction of Trained Immunity

To determine if milk and its major components can induce trained immunity, we screened the effects of raw milk, whey proteins, casein, milk fat, cream, and lactose, which were tested on monocytes using the experimental set-up, as depicted in Figure 1 (based on [32,33]). β-glucans were included as a positive control, as were TLR ligands as components that induce tolerance in this model system (see Supplementary Figure S2). As raw milk added directly to human monocytes reduced cell viability, raw milk’s major components were tested at the equivalent concentrations present in raw milk (casein, milk fat, cream, and lactose).

Supplementary Figure S2 shows that monocytes stimulated with β-glucans acted as a positive control, and when re-stimulated with R848, produced higher IL-6 and TNF-α levels compared to control cells (non-trained cells in culture medium). The same induction of IL-6 and TNF-α was also seen when cells trained with β-glucans were re-stimulated with Pam3CSK4 (Pam) and LPS. In contrast, training with TLR-ligands Pam, LPS, and R848 induced tolerance to re-stimulation, as published elsewhere [32,33], and was not due to cell death.

When raw milk and milk components were tested for their ability to induce trained immunity, raw milk induced variable production of IL-6 and TNF-α for different secondary stimulations, while inducing non-significant elevated levels of IL-6 upon Pam, LPS, and R848 stimulation compared to non-trained cells (Figure 2A). Whey proteins and cream induced trained immunity when re-stimulated with R848 (Figure 2A,B). This was also seen after stimulation with Pam and LPS. Milk fat was dissolved in Dimethyl sulfoxide (DMSO), which inhibited cytokine production in the assay. No additional induction of tolerance was observed by the milk fat diluted in DMSO, therefore it was not included in further experiments. As whey proteins induced trained immunity comparable to β-glucan for both IL-6 and TNF-α, the effect of three prominent immune-related whey proteins was investigated. LPS-free, isolated bovine lactoferrin (bLF) and bovine IgG (bIgG), but not Transforming Growth Factor (TGF)-β, seem to induce trained immunity. Bovine milk proteins are thus able to induce trained immunity.

### 3.2. Induction of Trained Immunity by Raw Milk and Bovine Immunoglobulin G

To extend and statistically analyse our initial observations, we studied the induction of trained immunity by raw milk, bIgG, and bLF in a number of additional individuals. β-glucan was included as a positive control. Stimulation of isolated monocytes with β-glucans consistently resulted in higher IL-6 production compared to non-trained monocytes upon re-stimulation with TLR-ligands Pam, LPS, and R848 (Supplementary Figure S3). Also, a higher TNF-α production by β-glucan trained monocytes was observed for LPS and R848 re-stimulated macrophages, but not for Pam re-stimulated macrophages.

**Figure 3:**
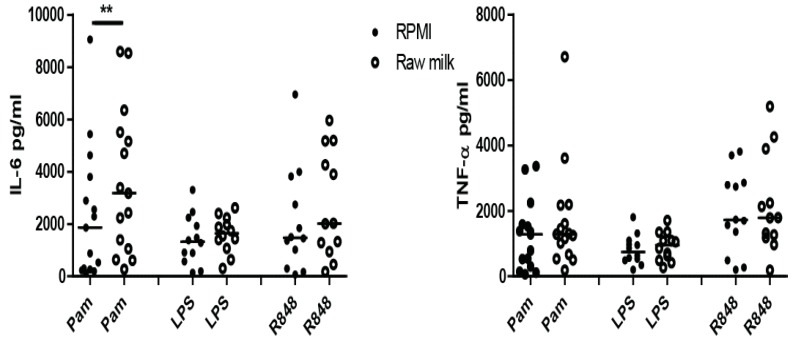
Induction of trained immunity by raw bovine milk. Monocytes were stimulated 24 h in the presence or absence of raw bovine milk (1:100); after five days of rest, the differentiated macrophages were re-stimulated for 24 h with Pam3Cysk4 (Pam) (10 μg/mL), Ultra-pure lipopolysaccharide (LPS) (0.1 μg/mL), or R848 (10 μg/mL). After stimulation with R848, the produced IL-6 and TNF-α (pg/mL) was measured in supernatant. In 5–6 independent experiments, *n* = 15 (Pam) or *n* = 12 (LPS, R848) Data shown as dot plot with median. Statistics was done by performing a Wilcoxon signed rank test between raw bovine milk and RPMI for every secondary stimulation (Pam, LPS, R848). ** *p* < 0.01.

Figure 3 shows that only upon re-stimulation with Pam, training with raw bovine milk is able to induce higher production of IL-6 compared to non-trained (RPMI) monocytes. This effect is not observed for IL-6 production after re-stimulation with other TLR-ligands, and not for TNF-α production after stimulation with any TLR-ligand (see also Supplementary Figure S4B).

**Figure 4:**
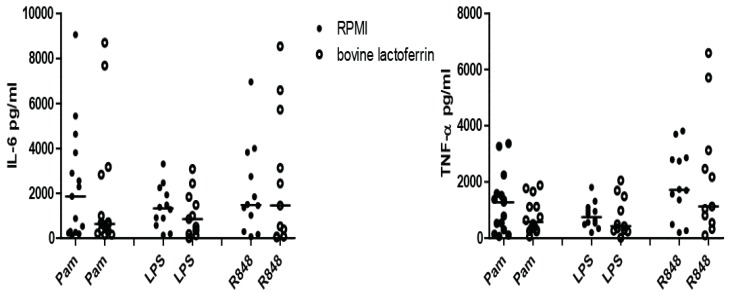
Bovine lactoferrin does not induce trained immunity. Monocytes were stimulated 24 h in the presence or absence of bovine lactoferrin (bLF) (100 μg/mL); after five days of rest, the differentiated macrophages were re-stimulated for 24 h with Pam3Cysk4 (10 μg/mL), LPS (0.1 μg/mL), or R848 (10 μg/mL). After stimulation with R848, the produced IL-6 and TNF-α (pg/mL) was measured in supernatant. In 5–6 independent experiments, *n* = 15 (Pam) or *n* = 11 (LPS, R848). Data shown as dot plot with median. Statistics was done by performing a Wilcoxon signed rank test between bLF and RPMI for every secondary stimulation (Pam, LPS, R848). No significant differences were observed.

In contrast to the screening experiment, bLF did not consistently induce trained immunity when more subjects were tested (see Figure 4). Supplementary Figure S4 and Supplementary Table S1 show that donors respond to bLF training in a very heterogeneous manner, in which only some donors show increased training by bLF, resulting in no statistically significant effect overall. 

**Figure 5:**
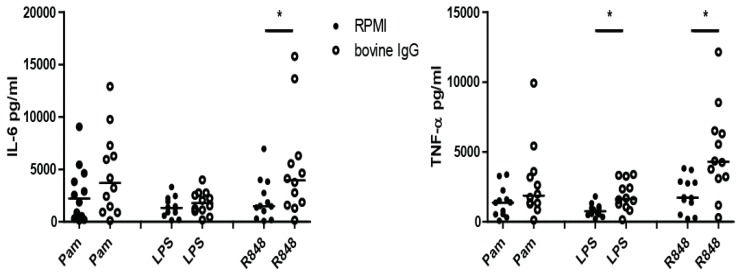
Induction of trained immunity by bovine IgG. Monocytes were stimulated 24 h in the presence or absence of bovine IgG (200 μg/mL); after five days of rest, the differentiated macrophages were re-stimulated for 24 h with Pam3Cysk4 (Pam) (10 μg/mL), LPS (0.1 μg/mL), or R848 (10 μg/mL). After stimulation with R848, the produced IL-6 and TNF-α (pg/mL) was measured in supernatant. In 5 independent experiments, *n* = 12. Data shown as dot plot with median. Statistics were done by performing a Wilcoxon signed rank test between bovine IgG and RPMI for every secondary stimulation (Pam, LPS, R848). * *p* < 0.05.

In line with the selection experiment and raw bovine milk, bovine IgG was able to consistently induce trained immunity (Figure 5). When monocytes were stimulated with bIgG and re-stimulated with R848, the cells produced more IL-6 and TNF-α and more TNF-α upon LPS stimulation compared to non-trained cells (RPMI). Upon re-stimulation with Pam, bIgG tended to increase the production of IL-6 and TNF-α, albeit not significantly (Supplementary Figure S4).

## 4. Discussion

Here we show that bIgG and raw bovine milk can induce trained immunity in human monocytes. These findings confirm the hypothesis that dietary components can modulate the responsiveness of the innate immune system to pathogen-related stimuli. 

One of the first components that was shown to induce trained immunity was β-1, 3-(d)-glucan derived from *C. albicans*, which exerted this effect via epigenetic changes in trimethylation of H3K4, induced after binding to Dectin-1 [15]. Epigenomic profile analysis revealed changes in H3K4m1, H3K4me3, and H3K27ac when comparing a β-glucan trained compared to non-trained macrophages [33]. Based on these studies β-1, 3-(d)-glucan derived from *C. albicans* has become a model compound to study the mechanisms of trained immunity.

Cell metabolism is important in monocyte-to-macrophage differentiation, and also for M1 versus M2 functions [36]. Resting and tolerant macrophages use oxidative phosphorylation to generate ATP, while activated (trained) macrophages shift to aerobic glycolysis (Warburg effect) via the dectin-1-Akt-mTOR and HIF-1α pathway [33,37]. Resting macrophages have a functional tricarboxylic acid (TCA) cycle that, together with glycolysis, enhances membrane synthesis and induces TLR-mediated activation in dendritic cells [33]. Two other metabolic pathways that are important in vitro and in vivo for trained immunity induction are glutaminolysis and the cholesterol synthesis pathway [38,39]. β-glucan-induced trained immunity via the dectin-1 pathway also effects the TCA cycle, as glycolysis inhibitors (rapamycin) also inhibit β-glucan-mediated trained immunity [38]. This indicates that β-glucan-induced trained immunity also effects the TCA cycle, and does not only signal via the dectin-1 pathway. This links cell metabolism, metabolites, and epigenetic mechanisms, suggesting that β-glucan induces trained immunity by modifying cell metabolism.

In our hands, β-glucan induced higher levels of IL-6 compared to non-trained cells when trained macrophages were re-stimulated with ligands for TLR1/2 (Pam), TLR4 (LPS), and TLR7/8 (R848). TNF-α production was increased after LPS and R848 re-stimulation. Unexpectedly, TNF-α production was not increased after Pam re-stimulation, which is in contrast to Ifrim et al., albeit using the same concentration and same supplier of Pam3CSK4 [32].

Until now, it has not been clear if dietary components can also induce immune training in humans after nutritional intervention. In a pilot study in humans, where baker’s yeast (*S. cerevisiae*)-derived β-glucans (1000 mg/day) were used as a food supplement for seven days, no trained immunity effects were observed [40]. In contrast, it was recently shown that diets high in fat can also induce trained immunity in vivo in mice, and oxLDL can induce immune training in human monocytes in vitro [17,18], suggesting that nutrition may indeed directly affect innate immune function. 

As breast milk and bovine milk contain many immune-modulating components [22], we set out to study whether (raw) bovine milk or milk components can induce trained immunity in human monocytes as well. A trained immunity effect was observed for IL-6 production by raw bovine milk when stimulated with TLR1/2 ligand PamCSK4, but not for stimulation through TLR4 or TLR7/8 (Figure 4). In contrast, treatment with bIgG induced trained immunity after stimulation of TLR4 (LPS) and TLR7/8 (R848), but not after TLR1/2 stimulation. This indicates that the training effect of raw milk (for TLR1/2) is not mediated via bLF or bovine IgG, as no effect was observed for training with bLF and bIgG after TLR1/2 stimulation. Furthermore, as undiluted and low dilutions of milk compromised viability of the monocytes, raw milk was tested at a 100-fold dilution, whereas bIgG was tested at the levels present in raw milk. This can explain why raw milk does not induce trained immunity via TLR7/8.

It is not clear which component in raw milk is responsible for this training effect. One possibility is that bovine miRNAs in extracellular vesicles (exosomes) may do this. These vesicles are described to induced higher production of IL-6, but not higher TNF-α production, in LPS stimulated RAW264.7 cells [41].

In our initial screen of raw milk and the major milk components, the clearest training effect after TLR7/8 stimulation was seen for whey proteins (Figure 2), as well as for the isolated whey proteins bLF and bIgG (data not shown). The whey proteins were a liquid whey fraction that contained 60% protein, and as with the raw milk, was used at a 100-fold dilution. However, it should be stated that this preparation also contained some casein and lactose. As these did not have an effect on innate immune training, we assume the effect seen using this whey proteins was the result of the presence of bIgG, and possibly also of bLF. Next to whey proteins, lipid-rich cream—but not milk fat (triglycerides)—induced a small training effect. This indicates that either the lipids or the intact milk fat globular membranes present in cream serum, but not the triglycerides, may induce trained immunity to some extent.

For bIgG a consistent trained immunity effect was observed for re-stimulation with TLR7/8. However, this was not seen for bLF; bLF could induce trained immunity in some donors (Supplementary Table S1, Supplementary Figure S4C), but the overall response was not significant (Figure 4). The lack of training induced by bLF in most donors was not due to cell death. Besides, in Supplementary Table S1 it can be observed that subjects in which no training for bLF is seen, trained immunity induction by other training-stimuli is possible. It can be thus concluded that the variability of trained immunity responses depends on the training stimulus, and the mechanisms responsible for this effect remain to be elucidated in future studies. Furthermore, we have recently observed that a three peripheral blood mononuclear cells—week dietary intervention with bLF could enhance the response of pDC of elderly women to TLR7/8 (van Splunter, unpublished observations), and future studies should determine the involvement of trained immunity in this effect.

BLF can be taken up by human cells via three different receptors: intelectin [42], low-density-lipoprotein (CD91) [43], and CXCR4 [43]. Most likely, bovine lactoferrin (LF) can also bind to soluble CD14 and TLR4, as human LF is 69% homologous to bovine LF and can bind sCD14 [44] and TLR4 [45], which are expressed by monocytes [46]. In a human study in which subjects received 200 mg bLF and 100 mg Ig-rich whey proteins twice per day, a significant reduction in rhinovirus induced common cold was observed [47]. This protective effect of bLF and bIgG on rhinovirus infections that are recognized by the immune system via TLR2 and TLR7/8 might thus be linked to trained immunity [48].

In addition to the effect shown after TLR7/8 re-stimulation, bIgG was also able to induce trained immunity when re-stimulated with TLR4, but not with TLR1/2. Bovine Immunoglobulin G is known to bind to human monocytes, macrophages, and monocyte-derived dendritic cells via FcyRII (CD32 receptor) [49]. Den Hartog et al. showed that bIgG could bind to human airway pathogens, such as respiratory syncytial virus (RSV), *haemophilus influenzae* type b (Hib) and influenza virus [49]. These pathogens can activate TLR2 and TLR4 (Hib and RSV), TLR7 (RSV and influenza) and TLR8 (RSV) [49,50,51].

We propose that the mechanism for the trained immunity induction by bIgG occurs via binding to FcyRII (CD32). In murine macrophages, crosslinking of FcyRII/III leads to activation of mitogen-activated protein (MAP) kinase family members p38 and JNK [52]. In human monocytes, inhibiting p38 and JNK abolished trained immunity induced by flagellin (TLR5) after re-stimulating through TLR4 [32]. TLR induced signaling via MyD88 or TIR-domain-containing adapter-inducing interferon-β (TRIF) leads to pro-inflammatory cytokine production, and is partly regulated by the p38MAPK/MK2 pathway [53,54]. Furthermore, in trained immunity epigenetic changes (e.g., H3K4 trimethylation) on the promotor of pro-inflammatory cytokine genes (IL-6, TNF-α) are induced by training stimuli, resulting in increased cytokine production [15]. Therefore, altogether, we hypothesize that bIgG can exert a trained immunity effect in macrophages by activating the MAP kinase pathway via FcyRII, thereby inducing epigenetic changes on the promotors of IL-6 and TNF-α genes, leading to enhanced NF-κB-dependent TLR-mediated responses. 

## 5. Conclusions

In summary, our data show that raw bovine milk and bIgG isolated from raw colostrum can induce trained immunity in human monocytes. This strengthens the hypothesis that diet can influence the responsiveness of the innate immune system. Important to underline however is that the trained immunity program induced by milk, and milk components are very likely different from those induced by other dietary components, such as the Western-type diet. This can be concluded by the deleterious effects of Western-diet-induced trained immunity on atherosclerosis, whereas no such effects have been reported for milk. Future whole-genome transcriptome and epigenome studies should describe the trained immunity activation program induced by milk, fully describing the impact of dairy milk on long-term reprogramming of innate immunity.

## Supplementary Materials

The following are available online at http://www.mdpi.com/2072-6643/10/10/1378/s1. Table S1: Trained immunity induced per training stimulus, compared to non-trained cells. after TLR7/8 (R848) stimulation (IL-6 production), Figure S1: Gating strategy and purity of isolated monocytes (84.6%). Staining was performed with live/death staining, CD3 and CD14, Figure S2: Induction of trained immunity or tolerance is dependent on the training of monocytes. Monocytes were stimulated 24 h in the presence or absence of β-glucan (1 μg/mL); Pam3Cysk4 (10 μg/mL), LPS (0.1 μg/mL) or R848 (10 μg/mL), after 5 days of rest the differentiated macrophages were stimulated for 24 h with R848 (10 μg/mL). After stimulation with R848 the produced IL-6 and TNF-α (pg/mL) was measured in supernatant. Data shown as mean ± SEM, with the IL-6 and TNF-α production of non-trained cells (RPMI) as *x*-axis. RPMI and β-glucan *n* = 5; TLR stimuli *n* = 3. Data was statistically analysed using a paired *t*-test. * *p* < 0.05, Figure S3: Induction of trained immunity by β-glucan. Monocytes were stimulated 24 h in the presence or absence of β-glucan (1 μg/mL), after 5 days of rest the differentiated macrophages were re-stimulated for 24 h with Pam3Cysk4 (Pam) (10 μg/mL), LPS (0.1 μg/mL) or R848 (10 μg/mL). After stimulation with R848 the produced IL-6 and TNF-α (pg/mL) was measured in supernatant. *n* = 15 (Pam) or *n* = 12 (LPS, R848) in 5–6 independent experiments. Data shown as dot plot with median. Statistics was done by performing a Wilcoxon signed rank test between beta-glucan and RPMI for every secondary stimulation (Pam, LPS, R848). * *p* < 0.05; ** *p* < 0.01, Figure S4; Paired analysis plots of monocytes trained with β-glucan, raw milk, bLF and bIgG and re-stimulated with Pam, LPS or R848 at day 6. In supernatant of day 7 IL-6 (pg/mL) was measured.
