# Supplementary material for: Induction of Trained Innate Immunity in Human Monocytes by Bovine Milk and Milk-Derived Immunoglobulin G
Source: Nutrients. 2018 Sep 27;10(10):1378. doi: 10.3390/nu10101378 (PMC6213000; doi:10.3390/nu10101378)
Supplement: Supplementary file 1 [file nutrients-10-01378-s001.pdf]

Supplementary

Table S1. Trained immunity induced per training stimulus, compared to non-trained cells. after TLR7/8 (R848) stimulation (IL-6 production).

donor	β-glucan	Raw milk	Bovine lactoferrin	Bovine IgG
1	+	+	+	+
2	+	+	+	+
3	+	-	+	+
4	+	+	0	+
5	+	+	-	+
6	-	0	-	-
7	+	0	0	+
8	+	+	-	+
9	+	+		+
10	+	-	-	-
11	+	+	-	+
12	0	-	-	-
Training	10/12	7/12	3/11	9/12

+: >10% higher IL-6 production of training stimulus compared to non-trained cells: trained immunity; -: <−10% lower IL-6 production of training stimulus compared to non-trained cells: tolerance. 0: comparable (−10 < % < 10%) IL-6 production of training stimulus compared to non-trained cells.

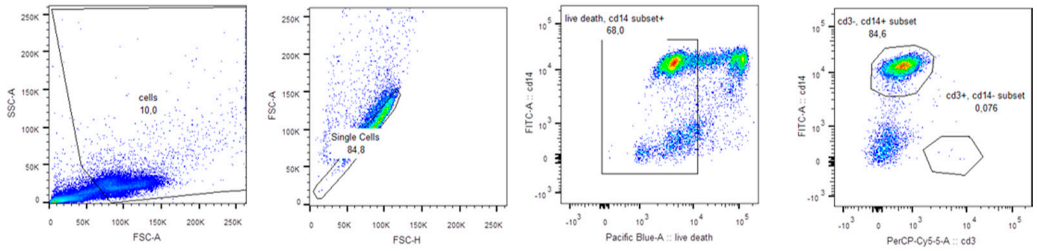

Figure S1. Gating strategy and purity of isolated monocytes (84.6%). Staining was performed with live/death staining, CD3 and CD14.

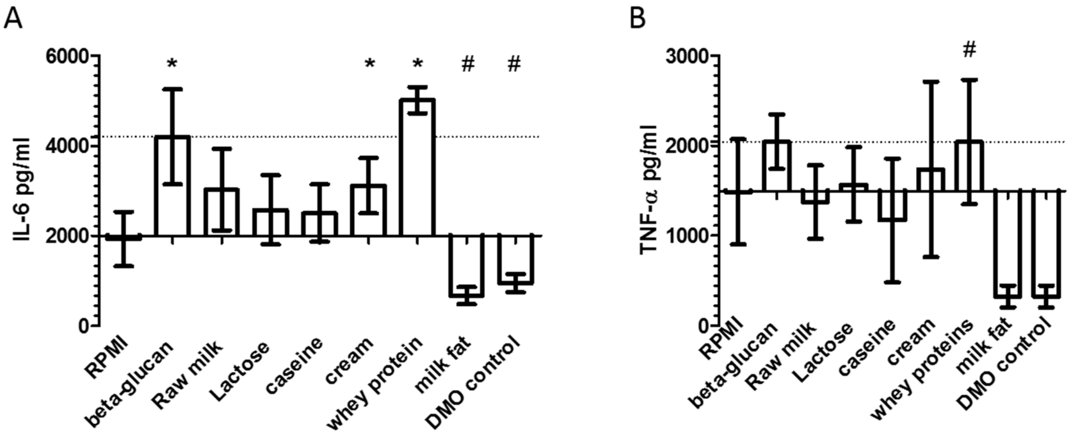

Figure S2. Induction of trained immunity or tolerance is dependent on the training of monocytes. Monocytes were stimulated 24 h in the presence or absence of β-glucan (1 μg/mL), Pam3Cysk4 (10 μg/mL), LPS (0.1 μg/mL), or R848 (10 μg/mL); after five days of

rest the differentiated macrophages were stimulated for 24 h with R848 (10 μg/mL. After stimulation with R848, the produced IL-6 and TNF-α (pg/mL) was measured in the supernatant. Data is shown as mean ± SEM, with the IL-6 and TNF-α production of non-trained cells (RPMI) as the x-axis. RPMI and β-glucan: $n = 5$; TLR stimuli: $n = 3$. Data was statistically analysed using a paired t-test. * $p < 0.05$,.

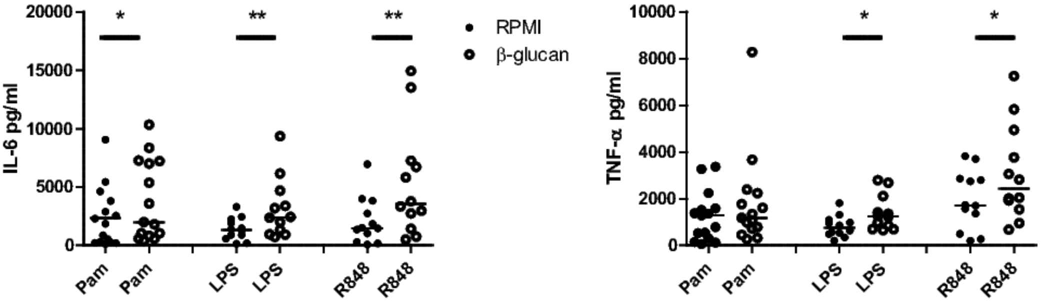

Figure S3. Induction of trained immunity by β-glucan. Monocytes were stimulated 24 h in the presence or absence of β-glucan (1μg/mL); after five days of rest the differentiated macrophages were re-stimulated for 24 h with Pam3Cysk4 (Pam) (10 μg/mL), LPS (0.1 μg/mL), or R848 (10 μg/mL). After stimulation with R848 the produced IL-6 and TNF-α (pg/mL) was measured in the supernatant. In 5–6 independent experiments, $n = 15$ (Pam) or $n = 12$ (LPS, R848). Data shown as dot plot with median. Statistics was done by performing a Wilcoxon signed rank test between beta-glucan and RPMI for every secondary stimulation (Pam, LPS, R848). * $p < 0.05$; ** $p < 0.01$.

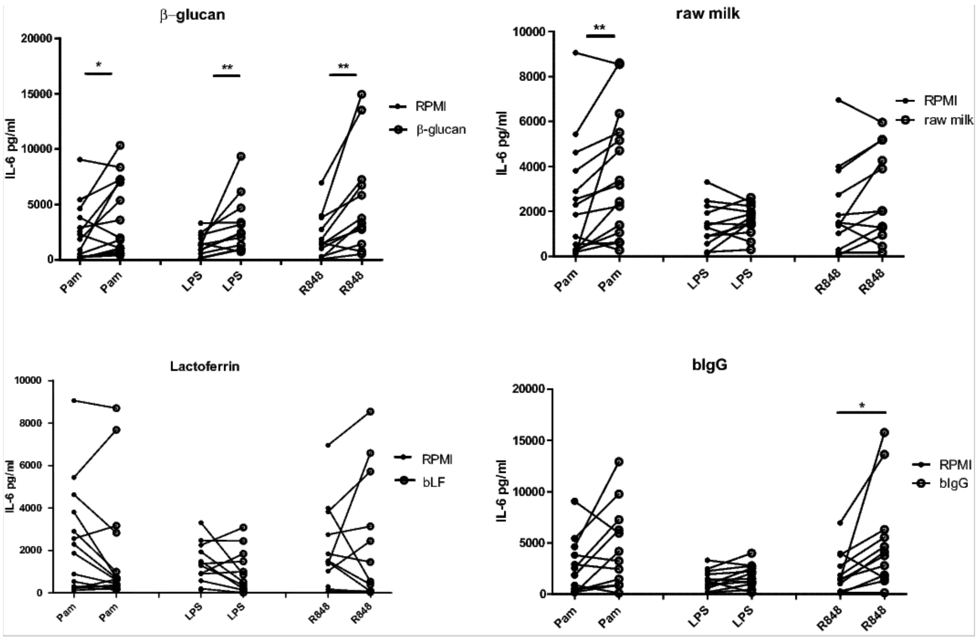

Figure S4. Paired analysis plots of monocytes trained with β-glucan, raw milk, bLF and bIgG, and re-stimulated with Pam, LPS, or R848 at day six. In the supernatant of day seven, IL-6 (pg/mL) was measured.
